# Case Report: Severe bilateral amyotrophic neuralgia associated with major dysphagia secondary to acute hepatitis E

**DOI:** 10.12688/f1000research.2-259.v2

**Published:** 2014-01-06

**Authors:** Xavier Moisset, Nicolas Vitello, Elodie Bicilli, Romain Courtin, Anna Ferrier, Frederic Taithe, Clément Lahaye, Ali Ait Hssain, Cyril Garrouste, Clavelou Pierre

**Affiliations:** 1 CHU Clermont-Ferrand, Service de Neurologie, CHU Gabriel Montpied, Clermont-Ferrand, F-63000, France; 2 Neuro-Dol, Inserm U1107, Douleur Trigéminale et Migraine, Faculté de Chirurgie Dentaire, F-63000, F-63000, France; 3 Clermont Université, Université d'Auvergne, Clermont-Ferrand, F-63000, France; 4 CHU Clermont-Ferrand, Service d’Ophtalmologie, CHU Gabriel Montpied, Clermont-Ferrand, F-63000, France; 5 CHU Clermont-Ferrand, Service de Rhumatologie, CHU Gabriel Montpied, Clermont-Ferrand, F-63000, France; 6 CHU Clermont-Ferrand, Service de Réanimation Médicale, CHU Gabriel Montpied, Clermont-Ferrand, F-63000, France; 7 CHU Clermont-Ferrand, Service de Néphrologie, CHU Gabriel Montpied, Clermont-Ferrand, F-63000, France

**Keywords:** Amyotrophic neuralgia, Hepatitis E, dysphagia

## Abstract

**Introduction:** Several acute neurological syndromes can be triggered by immune events. Hepatitis E virus (HEV), an emerging infectious disease, can be one of these triggers.

**Case report:** We report the case of a 36-year-old man that presented nausea and a dull abdominal pain for a week and then felt an acute neuralgic pain involving both shoulders that lasted for 8 to 10 hours. Immediately after, the patient presented a severe bilateral muscular weakness of the proximal part of both upper limbs, corresponding to an amyotrophic neuralgia. Two days after the shoulder pain, the patient presented a dysphagia necessitating tube feeding.  A blood sample confirmed hepatitis caused by hepatitis E virus (HEV; genotype 3F). Oral feeding resumed progressively after five months. The patient was fully independent for the activities of daily living but was still unable to work after six months.

**Conclusion:** Amyotrophic neuralgia and hepatitis E are both under-diagnosed. It is noteworthy that HEV can trigger amyotrophic neuralgia. Antiviral drugs, oral steroids and intravenous immunoglobulins can be proposed, but the optimal treatment has  not yet been determined.

## Introduction

Neurological syndromes such as Guillain-Barré Syndrome, transverse myelitis, encephalitis or amyotrophic neuralgia can be triggered by immune events. Hepatitis E virus (HEV), discovered in the 1980s, can be one of these triggers. Epidemics of hepatitis E occur periodically throughout the developing world, but autochthonous HEV infections have also been reported in most developed countries during the last decade. Several HEV-associated neurological syndromes have been described but are probably under-diagnosed ^1^.

## Case report

We report the case of a 36-year-old French man, Caucasian truck driver, without any significant medical history. The clinical symptoms started in May 2012 with nausea and a dull abdominal pain. No sign of chronic liver disease or of portal hypertension was noted. High liver enzymes were diagnosed after assay for: alanine aminotransferase (ALT) 1707 µmol/L (normal range: N<78), aspartate aminotransferase (AST) 554 µmol/L (N<37), gamma-glutamyltranspeptidase (GGT) 737 U/L (N<95) and alkaline phosphatase at 311 U/L (N<136). Total bilirubin level was at 54 µmol/L (N<17). There was no hepatitis A, B or C, no HIV and no sign of autoimmune disease. The immunological screening included antinuclear antibodies, anti-smooth muscles antibodies, anti-mitochondria antibodies, anti LKM antibodies, anti-hepatic cytosol antibodies, complement (C3, C4, CH50), rheumatoid factor, antineutrophil cytoplasmic antibody (ANCA), antiganglioside antibodies (GM1, GM2, GD1a, GD1b, GQ1b) and onconeuronal antibodies (Hu, Ri, Yo, PNMA2, CV2, Amphiphysine). Liver ultrasound was normal. The prothrombin time stayed within the normal range throughout the monitoring period.

Around one week after the first digestive symptoms, the patient felt an acute neuralgic pain involving both shoulders that lasted for 8 to 10 hours. Immediately after, the patient presented a severe bilateral muscular weakness of the proximal part of both upper limbs. Two days after the shoulder pain, the patient presented with hypophonia and dysphagia. The MRI did not show any brain abnormality. The spinal cord and the brachial plexus were unharmed. The cerebrospinal fluid (CSF) was normal (2 white blood cells/mm3; CSF Protein= 0.37 g/L) and there was no intrathecal antibody synthesis, although it was not tested for the synthesis of specific anti-HEV antibodies. Electromyography (EMG) and nerve conduction studies (NCS) showed normal amplitudes and conduction velocity but bilateral denervation in the supraspinatus, infraspinatus, subscapularis and deltoid muscles. An acute hepatitis E infection was suspected due to the presence of IgM and confirmed by PCR (genotype 3f). The initial serum HEV RNA count was 5.2 log-copies/ml. The PCR was negative in the CSF and was not initially performed in stools.

A treatment with intravenous immunoglobulins (Tegeline ^®^, LFB laboratory, France; 0.4 g/kg/day) was given for 5 days. Ribavirin (600 mg/day) was also introduced. Nine days after ribavirin initiation, the PCR showed 2.02 log-copies/ml and was negative after 18 days in both blood and stools. Ribavirin treatment was discontinued after 35 days. After three weeks, the patient still required nasogastric tube-feeding and a gastric feeding tube was placed endoscopically. There was no contraction of the shoulder girdle muscles. Oral feeding resumed progressively after five months. After six months follow-up and intensive rehabilitation, there was a 3/5 muscular strength in the affected muscles, corresponding to a movement possible against gravity, but not against resistance by the examiner. The patient was fully independent for the activities of daily living but still unable to work.

## Discussion

This is the first report of both severe bilateral amyotrophic neuralgia and dysphagia caused by an acute hepatitis E infection. Amyotrophic neuralgia (AN) is a peripheral neuropathy consisting of multiple symptoms including abrupt onset of shoulder pain, usually unilaterally, followed by motor weakness, with an annual incidence above 2 per 100,000 inhabitants ^2^. Concomitant involvement of other peripheral nervous system structures (such as the lumbosacral plexus or phrenic nerve) is described. AN can be triggered by immune events but also by trauma or surgery. Many patients are left with residual disabilities that affect their ability to work and their everyday life. It is noteworthy that a particularly severely affected subgroup presents sign of liver dysfunction, as seen in HEV infections ^2^. The only proposed treatment (with a low level of evidence) is corticosteroids but this may have been dangerous in this case of acute hepatitis E ^3^. Some authors have also reported a positive effect of intravenous immunoglobulins and this was the option we selected ^4– 6^.

HEV-associated neurological syndromes include both central and peripheral nervous system involvement ^7^. Such cases have been described in the Asian sub-continent (probably due to HEV1) but also in Western Europe with acute and chronic HEV3 infection. The present case concerned genotype 3f virus that is predominant in France ^8^. For patients with chronic HEV infections, neurological symptoms completely resolved or significantly improved when viral clearance was achieved ^1^. Moreover, several authors suggest treating severe acute HEV infections in order to preclude the development of acute liver failure ^9^. This is the reason why we tried antiviral treatment. Unfortunately, although viral clearance was achieved quickly, this did not lead to fast clinical improvements.

An alternative diagnosis of pharyngeal-cervical-brachial variant of Guillain-Barré syndrome could have been made. This pathology is characterized by oropharyngeal, neck, and upper limb muscle involvement. However, in the present case, this diagnosis was excluded (no neck involvement, atypical EMG, no albuminocytologic dissociation of the cerebrospinal fluid and negative GQ1b antibody).

## Conclusion

Post-infectious neurological diseases following HEV infection must be recognized to avoid unnecessary and potentially invasive procedures such as liver biopsy. Further studies are needed in order to determine the optimal treatment. In the meantime, antiviral drugs, steroids and IV-immunoglobulins are all possibilities.

## Consent

Written informed consent for publication of clinical details was obtained from the patient.
