# Supplementary material for: Case Report: Severe bilateral amyotrophic neuralgia associated with major dysphagia secondary to acute hepatitis E
Source: F1000Res. 2014 Jan 6;2:259. Originally published 2013 Nov 26. [Version 2] doi: 10.12688/f1000research.2-259.v2 (PMC3886787; doi:10.12688/f1000research.2-259.v2)
Supplement: Peer review history

# Peer review: Referee response for version 2

**DOI:** 10.5256/f1000research.3536.r4002

**Published:** 2014-03-07

**Authors:** Michelle Cheung

**Affiliations:** 1 King's College Hospital, London, UK

I have read this submission. I believe that I have an appropriate level of expertise to confirm that it is of an acceptable scientific standard.

# Peer review: Referee response for version 2

**DOI:** 10.5256/f1000research.3536.r3987

**Published:** 2014-03-06

**Authors:** Bruce Brew

**Affiliations:** 1 Department of Neurology, St Vincent's Hospital, Sydney, NSW, Australia

I have read this submission. I believe that I have an appropriate level of expertise to confirm that it is of an acceptable scientific standard.

# Peer review: Referee response for version 2

**DOI:** 10.5256/f1000research.3536.r3142

**Published:** 2014-01-16

**Authors:** Thierry Coton

**Affiliations:** 1 Service de Pathologie Digestive, Hôpital d’instruction des Armées Laveran, Marseille, France

This is a very interesting article fitted for a ‘letter to the editors' concerning emerging complications of HEV infection previously unknown to neurologists and gastroenterologists.

The title is appropriate for the content of the article and the abstract is a suitable summary of the text. The Bibliography is good quality, conclusions are clear and useful, all data are correct and missing ones are justified by the authors in the discussion.

This version can be accepted without corrections.

I have read this submission. I believe that I have an appropriate level of expertise to confirm that it is of an acceptable scientific standard.

# Peer review: Referee response for version 1

**DOI:** 10.5256/f1000research.2926.r2571

**Published:** 2013-12-23

**Authors:** Michelle Cheung

**Affiliations:** 1 King's College Hospital, London, UK

This is a report of an emerging viral infection with well-documented but still uncommon neurological symptoms, and adds to the knowledge of the natural history and potential treatment of this clinical syndrome. It is clearly written and the case is described well.

I have the following minor comments:

1. The referenced Cochrane review found no evidence from randomised trials to support any form of treatment for neuralgic amyotrophy, with only one retrospective series reporting a benefit. Therefore it seems an over-statement to say that corticosteroids are a validated treatment for the condition.
2. Are there any cases of prednisolone use specifically for HEV-induced neuralgic amyotrophy? What is the evidence of using ribavirin to treat acute (rather than chronic) HEV, which is a self-limiting infection?
3. The authors state that *'a severely affected subgroup present with signs of liver dysfunction, as seen in HEV*' - do you mean that cases associated with HEV infection (and therefore presenting with liver dysfunction) have a more severe clinical course compared to neuralgic amyotrophy associated with other causes?
4. Please justify the conclusion that recognition of HEV infection avoids invasive procedures?

I have read this submission. I believe that I have an appropriate level of expertise to confirm that it is of an acceptable scientific standard.

# Response

**Published:** 2013-12-31

**Authors:** Xavier Moisset

**Affiliations:** Centre de Traitement et d'Evaluation de la Douleur, CHU Ambroise Paré, France

We want to thank Dr Cheung for the comments she made that have helped us to improve the quality of our text.

1.  Dr Cheung is right; the Cochrane review identified one open label retrospective series suggesting that prednisone can shorten the duration of the initial pain and leads to earlier recovery in some patients. Thus, the expression “validated treatment” has been replaced by “ *proposed treatment (with a low level of evidence)*”.
2. To our knowledge, there is no description of prednisone use in the specific case of HEV-induced neuralgic amyotrophy. Concerning the use of ribavirin to treat acute HEV, there is a low level of evidence but several authors suggest treating severe acute form in order to preclude the development of acute liver failure (Abbas Z and Afzal R. 2013 [Epub ahead of print] Hepatitis E: when to treat and how to treat). A sentence and this reference are now included in the discussion section.
3. We cannot know if neuralgic amyotrophy secondary to HEV infections are more severe compared with neuralgic amyotrophy associated with other causes. But among the patients with amyotrophic neuralgia, it has been described that a subgroup of patients had a particularly severe clinical course and that these patients had liver dysfunction. Thus, we hypothesized that some of these patients were possibly affected by HEV.
4. Unexplained severe hepatic cytolysis can lead to liver biopsy. HEV infection recognition can avoid this invasive procedure.

# Peer review: Referee response for version 1

**DOI:** 10.5256/f1000research.2926.r2868

**Published:** 2013-12-20

**Authors:** José Manuel Echevarría

**Affiliations:** 1 Department of Virology, National Centre of Microbiology, Carlos III Health Institute Majadahonda, Madrid, Spain

The manuscript describes the association of a convincingly diagnosed case of acute hepatitis E due to HEV genotype 3 infection with the development of severe amyotrophic neuralgia and major dysphagia in the patient. Reports of neurological complications among otherwise healthy patients after acute HEV-3 infection are scarce (I found just six cases from three reports since 2009, five of them reported in 2011), and the manuscript adds significant knowledge to the background.

Pathogenic mechanisms underlying neurological complications of hepatitis E are still unclear. Such diseases after viral infection may involve the invasion of the nervous system or may be of a pure, post-infectious, immune-mediated nature. Reports involving immunocompromised patients ( Kamar *et al.*, 2011; four patients) found HEV genome in CSF from all of them and detected anti-HEV IgM in one, which confirmed CNS invasion at the time of the onset of the neurological symptoms.

Among the six healthy patients mentioned above ( Loly *et al.,* 2009; Kamar *et al.,* 2011; *Despierres et al., *2011), CSF testing for HEV markers was performed in three cases. One tested positive for both HEV RNA and anti-HEV IgM, and one tested negative for both markers ( Despierres *et al., *2011). CSF from the remaining patient was tested for viral RNA only and was found negative. It seems likely therefore, that both mechanisms may be involved, in absence of immune impairment.

The patient mentioned in the present report tested negative for HEV RNA in CSF, but I understand from the text that anti-HEV testing of CSF was not performed (if the sentence “ *there was no intrathecal antibody synthesis*” refers to immunoglobulins and not to HEV-specific antibody, which should be stated more clearly). It would have provided useful information if the patient’s CSF had been tested for anti-HEV IgG and IgM. The lack of a viral genome at the time of sampling does not exclude CNS invasion, and demonstration of intrathecal synthesis of a specific antibody provides the diagnosis once viral particles are no longer present. As far as I know, nobody has yet communicated results from the evaluation of the intrathecal synthesis of a specific antibody to HEV in these cases (only the presence of anti-HEV IgM has been documented), so I would suggest performing such an evaluation if anti-HEV was detected. From my former experience with the diagnosis of neurological infections caused by varicella-zoster virus, testing for specific IgG antibody optimizes the yield, and the most useful criteria of evaluation is the antibody to albumin index, though several others can be used (see Echevarría *et al. *1997).

I have read this submission. I believe that I have an appropriate level of expertise to confirm that it is of an acceptable scientific standard.

# Response

**Published:** 2013-12-31

**Authors:** Xavier Moisset

**Affiliations:** Centre de Traitement et d'Evaluation de la Douleur, CHU Ambroise Paré, France

Dr Echevarría is right; anti-HEV antibody testing of CSF was not performed and this is now clearly specified in our version 2. Indeed, it would be of interest in future cases to test CSF for both RNA and intrathecal synthesis of specific anti-HEV antibodies.

# Peer review: Referee response for version 1

**DOI:** 10.5256/f1000research.2926.r2575

**Published:** 2013-12-20

**Authors:** Thierry Coton

**Affiliations:** 1 Service de Pathologie Digestive, Hôpital d’instruction des Armées Laveran, Marseille, France

This is a very interesting article about an emerging complication of acute hepatitis E.

The following information is missing however:

1. the gender of the patient.
2. if prothrombin time increased during hepatitis.
3. which auto antibodies were screened for.
4. if PCR was realised in stools.
5. the duration of the ribavirine therapy.

Additionally in the discussion, the authors must highlight that this case concerned a genotype 3f virus which is predominant in France ( Luciano *et al.* 2012).

I have read this submission. I believe that I have an appropriate level of expertise to confirm that it is of an acceptable scientific standard.

# Response

**Published:** 2013-12-31

**Authors:** Xavier Moisset

**Affiliations:** Centre de Traitement et d'Evaluation de la Douleur, CHU Ambroise Paré, France

We want to thank Dr Coton for the comments and suggestions he made that have helped us to improve the quality of our case report.

1. The gender of the patient (male) was specified in the abstract but not in the core text. It is now corrected.
2. The prothrombin time stayed stable within the normal range throughout the monitoring period.
3. The immunological screening included: antinuclear antibodies, anti-smooth muscles antibodies, anti-mitochondria antibodies, anti LKM antibodies, anti-hepatic cytosol antibodies, complement (C3, C4, CH50), rheumatoid factor, antineutrophil cytoplasmic antibody (ANCA), anti ganglioside antibodies and onconeuronal antibodies (Hu, Ri, Yo, PNMA2, CV2, Amphiphysine).
4. PCR was not initially realized in stools. The only PCR in stools was realized after 3 weeks of Ribavirin treatment and was negative.
5. The ribavirin therapy lasted for 35 days. The treatment was stopped on the basis of negative PCR results in both blood and stools.
6. We now specify in the discussion that this case concerned a genotype 3f virus which is predominant in France.

# Peer review: Referee response for version 1

**DOI:** 10.5256/f1000research.2926.r2574

**Published:** 2013-12-04

**Authors:** Bruce Brew

**Affiliations:** 1 Department of Neurology, St Vincent's Hospital, Sydney, NSW, Australia

This is an important case report highlighting HEV as a cause for brachial neuritis. Recognition of this association is clinically important as treatment with corticosteroids should be avoided.

I have read this submission. I believe that I have an appropriate level of expertise to confirm that it is of an acceptable scientific standard.
